# Early Radiological Intervention in Budd–Chiari Syndrome: Clinical Outcomes From a Single‐Center Study

**DOI:** 10.1155/ijh/2141081

**Published:** 2026-08-12

**Authors:** Tai Ermongkonchai, Alysia Bastas, Katrina Tan, Adam Testro

**Affiliations:** ^1^ Liver Transplant Unit Victoria, Austin Health, Heidelberg, Victoria, Australia, austin.org.au; ^2^ Australian Centre for Transplantation Excellence and Research, Heidelberg, Victoria, Australia; ^3^ Department of Surgery (Austin Precinct), The University of Melbourne, Melbourne, Australia, unimelb.edu.au

**Keywords:** Budd–Chiari syndrome, liver transplant, myeloproliferative disorder, transjugular intrahepatic portosystemic shunt

## Abstract

**Background/Aims:**

Budd–Chiari Syndrome (BCS) is a vascular disease of the liver characterized by venous outflow obstruction most often due to a myeloproliferative neoplasm (MPN). Restoration of hepatic venous outflow and long‐term anticoagulation remain the mainstay of treatment. We examined our experiences and treatment outcomes of BCS, including comprehensive testing for underlying MPN.

**Methods:**

A retrospective study was conducted to assess overall and transplant‐free survival in patients with primary BCS. All cases of primary BCS that presented to our institution over the past 3 decades were included. Details relating to their hematological work‐up and radiological interventions were recorded, as well as their longer term outcomes, including the need for liver transplantation.

**Results:**

Forty‐three primary BCS patients were identified over a 28‐year period, with a median follow‐up time of 134 months. MPN was detected in 25 (62.5%) of cases who had comprehensive testing. All patients received primary anticoagulation. Seventeen (39.5%) patients underwent primary transjugular intrahepatic portosystemic shunt (TIPS). Twenty (46.5%) received anticoagulation alone as primary therapy, of whom 14 (70%) failed therapy and ultimately needed a rescue TIPS. Four (9.3%) patients underwent liver transplantation. Overall survival was 100% and 92.9% at 1 and 5 years, respectively, with transplant‐free survival of 97.7% and 88.1% at 1 and 5 years, respectively.

**Conclusion:**

MPN (particularly JAK2V617F‐positive) is an important underlying cause of primary BCS. Anticoagulation alone without radiological intervention can lead to disease progression. In this single‐center cohort, early TIPS for rapid hepatic decompression appeared safe and was associated with excellent long‐term outcomes.

## 1. Introduction

Budd–Chiari Syndrome (BCS) is a rare vascular disease of the liver characterized by hepatic venous outflow obstruction, with a global incidence rate of 1.0 per million annually [1, 2]. Primary BCS is an intrinsic venous disorder that occurs most commonly from thrombosis of the hepatic vein and inferior vena cava (IVC). Secondary BCS results from extraluminal venous compression due to intra‐abdominal abscesses or tumors [3].

An identifiable prothrombotic risk factor for primary BCS is found in approximately 75% of patients, with myeloproliferative neoplasms (MPNs) being the most common, followed by thrombophilia (congenital or acquired) and systemic hormone replacement therapy [1]. No risk factors are found in 15%–30% of patients. Primary BCS may present in patients with a previously diagnosed MPN or can occasionally be the first presentation of a previously undiagnosed MPN [4]. A meta‐analysis of 1062 BCS patients found that MPN was prevalent in 41% of patients; however, this likely represents a significant underestimation as only 38% of these patients completed clinical and laboratory diagnostic testing for MPN, including JAK2V617F mutation testing, where there is 95% and 50% positivity in patients with polycythemia rubra vera (PRV) and essential thrombocythemia (ET), respectively [4, 5].

The treatment goals of BCS are threefold: (i) restoration of hepatic venous outflow, which in turn improves hepatic sinusoidal perfusion and function; (ii) management of portal hypertensive complications; and (iii) long‐term management of the underlying hematological disorder. There is a range of multidisciplinary therapeutic interventions commonly offered to patients with primary BCS, either sequentially (step‐up) or simultaneously. These include anticoagulation therapy (with vitamin K antagonists such as warfarin, a low–molecular‐weight heparin [LMWH] and/or a direct‐acting oral anticoagulant [DOAC]), percutaneous radiological interventions, such as hepatic venoplasty/stenting and transjugular intrahepatic portosystemic shunt (TIPS), or a modified portosystemic shunt if there is an unsuccessful TIPS insertion, with liver transplantation (LT) reserved as a final rescue therapy [6–8]. A study assessing a sequential treatment algorithm demonstrated 96% and 89% survival at 1 and 5 years, respectively, with 22% of their cohort escalating to LT [6]. Both AASLD and EASL clinical practice guidelines support the use of a step‐up strategy, starting with medical therapy and culminating in LT [9, 10]. However, our local practice has favored an early escalation to TIPS as primary therapy in conjunction with anticoagulation, to rapidly decompress the liver and thus facilitate earlier recovery of hepatic function and minimize the long‐term complications of portal hypertension [11].

Diagnostic genetic testing for MPN, including JAK2V617F gene testing and the calreticulin receptor (CALR) mutation testing has become routine standard‐of‐care over the past decade. Up to 80% of JAK2V617F‐negative MPN patients are positive for the CALR gene mutation [12]. This allows for early identification of genetic risk factors that predispose to MPN and primary BCS.

The aim of this study is to report our experiences and treatment outcomes of managing primary BCS with early radiological intervention, including comprehensive testing for underlying risk factors.

## 2. Methods

### 2.1. Study Design

A retrospective observational study was conducted at Austin Health, Australia, to assess overall and transplant‐free survival in patients with primary BCS. All cases of primary BCS that presented to our institution over the past 3 decades (March 1994 to February 2023) were included in this study. The start date of the study was chosen to reflect when the polytetrafluoroethylene (PTFE) covered stents were introduced at our institution. Patients with secondary BCS were excluded from the analysis. This study was approved by the local ethics committee (HREC/97834/Austin‐2023).

### 2.2. Data Collection

Patients were identified from the hospital electronic and scanned medical records. All data, including patient demographics, clinical presentation, investigations, treatment, and outcomes, were obtained. Genetic testing panels to identify underlying risk factors in MPNs and thrombophilia were also collected. MPN screening tests involve peripheral cell counts, blood film, and gene mutation testing (JAK2V617F and CALR testing in JAK2‐negative cases). Thrombophilia testing consists of Factor V Leiden and prothrombin gene mutations, antiphospholipid antibodies, protein C and S deficiencies, antithrombin deficiency, homocysteine levels, and paroxysmal nocturnal hemoglobinuria (PNH) flow cytometry. Clinical prognostic scores such as the Model for End‐Stage Liver Disease (MELD), Rotterdam, and TIPS‐BCS prognostic index scores were calculated from the clinical data obtained.

We defined primary treatment as the initial therapy/intervention commencing within 3 months of BCS diagnosis, with secondary treatment indicated in the case of primary treatment failure. Treatment failure was defined as either clinical (e.g., worsening ascites or signs of portal hypertension), biochemical (worsening prognostic scores), and/or radiological evidence of progression while on treatment. The decision on upfront TIPS/direct intrahepatic portocaval shunt (DIPS) as primary management was determined on an individual basis following clinical review by hepatology and interventional radiology based on each patient′s clinical presentation, biochemical markers, and prognostication scores.

### 2.3. Prognostication Scores

The MELD score, derived by the Cox proportional hazards regression model, was originally a prognostication tool that uses serum bilirubin, creatinine, and international normalized ratio (INR) to predict mortality at 3 months in cirrhotic patients having elective TIPS procedures [13]. Due to its accuracy in predicting short‐term survival in cirrhosis, it is now one of the most commonly used prognostic tools for cirrhosis, including guiding the triaging and prioritization of cases for LT. Higher MELD scores indicate poorer survival rates at 3 months: 1.9% mortality with MELD < 11, 6% mortality with MELD 11–19, 19.6% mortality with MELD 20–29, 52.6% mortality with MELD 30–39, and 71.3% mortality with MELD ≥ 40 [14], with high mortality after TIPS procedures [15]. We calculated and recorded MELD scores at the time of BCS diagnosis and at the time of the first radiological procedure (venoplasty, stenting, or TIPS) to assess the progression of liver disease.

The Rotterdam score was calculated for all patients and predicts 3‐month mortality postdiagnosis, assisting with triaging the urgency of treatment [16]. It is calculated using the equation 1.27  ×  encephalopathy  +  1.04  ×  ascites  +  0.72  × prothrombin time  +  0.004 × bilirubin, where encephalopathy and ascites are binarily represented as 0 (absent) or 1 (present), and prothrombin time as 0 (INR < 2.3) or 1 (INR > 2.3) [17]. Patients are classed into three groups according to their Rotterdam scores: Class I are scores less than 1.1 (good prognosis), Class II are scores between 1.1 and 1.5 (intermediate prognosis), and Class III are scores over 1.5 (poor prognosis) [17].

The BCS‐TIPS prognostic index score (TIPS‐BCS PI score) is a risk stratification index that has been shown to predict LT‐free survival in patients with TIPS. It is calculated according to the following formula: age (years) × 0.08 + bilirubin (mg/dL) × 0.16 + INR × 0.63 [18], where a TIPS‐BCS PI score of over 7 predicts the need for LT or death at 1 year post‐TIPS insertion (58% sensitivity, 99% specificity) [19]. We calculated a TIPS‐BCS PI score for all patients in our cohort that received TIPS.

### 2.4. Outcome Measures

Overall survival (months) was determined as the time from diagnosis to death, or until the end of the follow‐up period (October 1, 2023). Transplant‐free survival was the total time the patient remained LT‐free from BCS diagnosis to death or until the end of follow‐up. 1‐ and 5‐year survival rates were calculated from the number of patients remaining alive at the specified follow‐up time divided by the cohort number. Patients lost to follow‐up were removed from these calculations from the time of their last known follow‐up. From a hepatology perspective, decompensated liver disease was defined as presence of hepatic encephalopathy, ascites, and/or jaundice. Due to the routine use of therapeutic anticoagulation, coagulopathy was not included when assessing hepatic dysfunction.

### 2.5. Statistical Analysis

Data are expressed as basic descriptive statistics with quantitative values (e.g., median and range) and qualitative values (absolute and relative frequencies).

## 3. Results

### 3.1. Patient Demographics

A total of 43 primary BCS patients were included in the study from the 28‐year period, with a median follow‐up time of 134 months (range 12–277 months). Out of the 43 patients, 7 had a pre‐existing diagnosis of BCS prior to presenting to our service for the first time with recurrent disease, whereas the remainder (*n* = 36) were newly diagnosed cases. Eight patients were lost to follow‐up at 38, 42, 47, 127, 183, 214, 220, and 244 months, respectively, and thus were censored at the last known follow‐up. (Table 1).

**Table 1:** Demographics of the patients with primary Budd–Chiari syndrome (*n* = 43).

Age of diagnosis (years): median (range)	43 (17–76)
Male gender (*n*)	16 (37.2%)
MELD score at diagnosis: median (range)	14 (6–29)
Number of patients with MELD score ≤ 17	22
Number of patients with MELD score ≥ 18	11
Number of patients where MELD score not calculated due to insufficient initial data	10
Rotterdam score: median (range)	1.13 (0.07–2.11)
TIPS‐BCS PI score at the time of first TIPS: median (range)	5.1 (2.5–6.8)
Number of patients in each Rotterdam class (I/II/III)	16 (37.2%)/18 (41.9%)/5 (11.6%)
Number of patients where Rotterdam score not calculated due to insufficient initial data	4
Concurrent portal vein thrombosis (*n*)	14 (32.6%)

Abbreviations: BCS, Budd–Chiari syndrome; MELD, Model for End‐Stage Liver Disease; PI, prognostic index; TIPS, transjugular intrahepatic portosystemic shunt.

Thirty‐eight (88.4%) patients presented with clinical symptoms of BCS, with ascites and abdominal pain being the most common (28 [65.1%] and 15 [34.9%], respectively). Three of these patients presented with acute liver failure. One patient had BCS diagnosed incidentally during a workup to investigate postviral thrombocytopenia. Fourteen patients (32.6%) had concurrent portal vein thrombosis (PVT). The median Rotterdam score was 1.13 (range 0.07–2.11). Thirty patients had TIPS inserted, with another two requiring a DIPS. The median TIPS‐BCS PI score was 5.1 (range 2.5–6.8).

### 3.2. Risk Factors for BCS

#### 3.2.1. MPN

Forty (93%) patients had investigations for MPN. JAK2V617F mutation molecular testing was the most common investigation for MPN (*n* = 34), followed by bone marrow biopsy (*n* = 17). Of the three remaining patients, two died prior to the availability of JAK2V617F mutation molecular testing (one due to end‐stage cirrhosis and the second due to intracranial hemorrhage), whereas one had other identifiable risk factors (Behçet′s disease and prothrombin gene mutation).

Of the 40 patients tested, 25 (62.5%) had MPN detected. Ten of these patients (25%) were diagnosed via JAK2V617F gene mutation positivity alone, 11 patients (27.5%) via JAK2V617F positivity and a confirmatory bone marrow biopsy, and 4 patients (10%) through bone marrow biopsy alone. Twelve patients were tested for the CALR mutation, with two (16.7%) testing positive—one patient with JAK2 negative MPN and the other case with ET of unknown JAK2 mutation status. The most common MPN subtype detected was PV, consisting of 14 cases (where two of these patients later transformed into myelofibrosis). Other MPN subtypes detected were ET (*n* = 6), MPN unclassified (*n* = 4), and one patient with chronic myeloid leukemia (CML) with concurrent JAK2V617F positivity.

**Figure 1:**
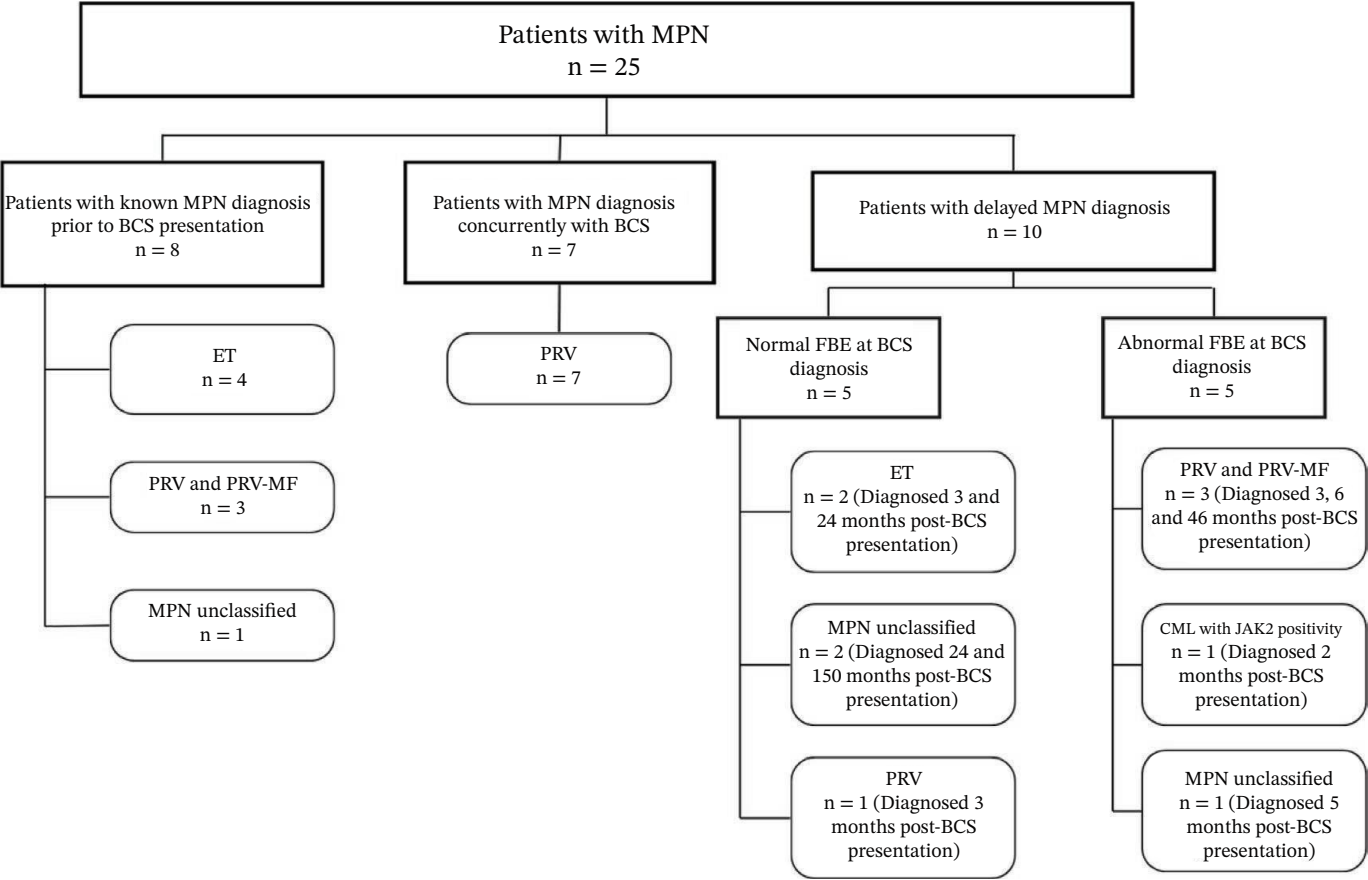
Subtypes of myeloproliferative neoplasms and time of diagnoses relative to BCS presentation. Abbreviations: BCS, Budd–Chiari syndrome; CML, chronic myeloid leukemia; ET, essential thrombocythemia; FBE, full blood examination; MPN, myeloproliferative neoplasm; PRV, polycythemia (rubra) vera; PRV‐MF, polycythemia (rubra) vera–myelofibrosis.

The MPN subtypes and diagnoses relative to the time of BCS presentation are displayed in Figure 1. Five patients had normal full blood examination (FBE) during BCS diagnosis, leading to a delayed diagnosis of MPN. The median hemoglobin at diagnosis for patients with MPN was 134 g/L (range 97–201 g/L), compared with a median of 131 g/L (range 108–181 g/L) in those without MPN. The median platelet count at diagnosis for patients with MPN was 346 × 10^9^/L (range 209–1099 × 10^9^/L), compared with a median of 268 × 10^9^/L (range 132–638 × 10^9^/L) in those without MPN.

#### 3.2.2. Other Risk Factors

Other risk factors for BCS identified in the patient cohort (with and without MPN) are displayed in Table 2.

**Table 2:** Additional risk factors for BCS.

Risk factors	MPN patients (number tested)	Non‐MPN patients (number tested)
Antiphospholipid antibodies	1/14	1/14
Antithrombin deficiency	1/9	2/13
Factor V Leiden mutation heterozygosity	3/14	4/14
Hyperhomocysteinemia	0/5	1/7
Estrogen therapy (oral contraceptive pill, in vitro fertilization, or hormone replacement therapy)	6/16	2/11
PNH	0/4	0/8
Protein C deficiency	4/13	8/14
Protein S deficiency	1/13	2/13
Prothrombin gene mutation	0/12	0/13
Alcoholic liver disease	1	0
Behçet′s disease	0	1
Crohn′s disease	0	2
Sarcoidosis	0	2

Abbreviations: MPN, myeloproliferative neoplasm; PNH, paroxysmal nocturnal hemoglobinuria.

#### 3.2.3. Distribution of Thrombosis

Twenty‐five (58.1%) patients had isolated large hepatic vein thrombosis, whereas 16 (37.2%) patients had both large hepatic vein and IVC involvement. Two patients had unknown distribution due to unavailable diagnostic data from other health services. Fifteen patients (34.9%) had concurrent PVT with BCS.

### 3.3. Medical Treatment and Interventions

#### 3.3.1. Hematological Management

All patients were anticoagulated with either warfarin (*n* = 37), LMWH (enoxaparin) (*n* = 19), or DOAC (apixaban or rivaroxaban) (*n* = 14), with 22 (51.2%) patients having more than one type of anticoagulation throughout their treatment. Overall, warfarin was the most prescribed anticoagulant across the entire study period, but from 2012 onwards, there was increased use of DOACs, including the transition of patients from warfarin to apixaban or rivaroxaban.

Ten (23.2%) patients experienced 13 episodes of major bleeding, with 11 of these episodes (in eight patients) occurring while on anticoagulation. Only three episodes of bleeding were in the setting of supratherapeutic anticoagulation (INR [> 3.0] on warfarin or anti‐Xa levels [> 1.0 taken at least 4 h postdose] on enoxaparin). Six (46.2%) episodes of bleeding were due to esophageal variceal hemorrhage. There were three episodes of intracranial hemorrhage, which were fatal in all cases.

Of the eight patients with a pre‐existing diagnosis of MPN, only one (12.5%) was on therapeutic anticoagulation (rivaroxaban) at the time of BCS diagnosis. Four (50%) were receiving other treatment such as aspirin, hydroxyurea, or venesection. In addition to routine anticoagulation in all 17 patients with newly diagnosed MPN, other therapeutic treatment consisted of hydroxyurea alone (*n* = 5), venesection alone (*n* = 2), hydroxyurea and venesection (*n* = 2), hydroxyurea and splenectomy (*n* = 1), hydroxyurea and aspirin (*n* = 2), hydroxyurea with concurrent aspirin and venesection (*n* = 2), and interferon (*n* = 1). Three patients with MPN were not commenced on cytoreductive treatment, two of which were due to normal blood counts and one having their diagnosis confirmed just before death. There was no MPN treatment documented for one patient.

#### 3.3.2. Hepatological Management

Thirty‐eight (88.4%) patients had radiological interventions during their BCS management, which was often multimodal—30 (69.8%) patients had TIPS inserted and 27 (62.8%) patients had at least one angioplasty/stent intervention. Due to large clot burden, two patients required a DIPS as an alternative to TIPS, meaning that 32 (74.5%) patients required a TIPS/DIPS as treatment.

Table 3 represents the distribution of the timing to intervention. Twenty (46.5%) patients were commenced on anticoagulation alone, 6 (14%) patients had anticoagulation with additional angioplasty and/or stenting, and 17 (39.5%) patients were treated with anticoagulation and upfront TIPS/DIPS as primary management. The median MELD score for patients with primary TIPS/DIPS was 18.5 (range 9–29), compared with a median MELD of 10.5 (range 6–20) among those without primary TIPS/DIPS. The primary TIPS/DIPS group had 5 (29.4%), 10 (58.8%), and 2 (11.8%) patients in Rotterdam Classes I/II/III, respectively, whereas the remaining cohort had 11 (42.3%), 8 (30.7%), and 3 (11.5%) patients in Rotterdam Classes I/II/III, respectively. Four patients had insufficient data to calculate their Rotterdam scores.

**Table 3:** Distribution of patients receiving primary therapy and/or escalation to secondary therapy with TIPS/DIPS.

Primary treatment	*n* (%), median (range)

Anticoagulation alone or with angioplasty/stenting (*n*)	26
MELD score at diagnosis: median (range)	‐10.5(6–20)
Number of patients in each Rotterdam class (I/II/III)	‐11 (42.3)/8 (30.7)/3 (11.5)
Anticoagulation + TIPS/DIPS (*n*)	17
MELD score at diagnosis: median (range)	‐18.5 (9–29)
Number of patients in each Rotterdam class (I/II/III)	‐5 (29.4)/10 (58.8)/2 (11.8)

Secondary treatment	*n* (%), median (range)

TIPS/DIPS (*n*)	14
Failed anticoagulation alone	
TIPS/DIPS (*n*)	1
Failed anticoagulation + angioplasty/stenting	

Abbreviations: DIPS, direct intrahepatic portocaval shunt; TIPS, transjugular intrahepatic portosystemic shunt.

Of the 26 (60.5%) patients that did not receive a primary TIPS/DIPS, 15 (57.8%) required secondary TIPS/DIPS due to failure of primary therapy at a median time of 15 months post‐BCS diagnosis (range 3–123 months). Fourteen (70%) patients who received primary anticoagulation alone subsequently required second line TIPS/DIPS.

All patients that received a TIPS/DIPS underwent a surveillance program with 6‐monthly Doppler ultrasound, where a venogram was indicated if there were abnormalities detected on ultrasound. Nineteen (59.4%) patients developed TIPS dysfunction, requiring a median of four revisionary interventions (range 1–14).

#### 3.3.3. LT

Four (9.3%) patients underwent LT. No patients required LT as a primary therapy for liver failure related to BCS alone. However, one patient required urgent transplantation 3 months postdiagnosis after developing acute liver failure as a complication resulting directly from an attempted DIPS at a different health center. Two patients underwent LT at 48 and 120 months, respectively, for chronic TIPS dysfunction resulting in portal hypertension and progressive hepatic decompensation. One patient, who had not required a TIPS/DIPS in management of BCS, had an LT at 169 months due to development of hepatocellular carcinoma. All four transplant patients were alive with stable graft function at the conclusion of the study period.

### 3.4. Outcome and Survival

Overall survival was 100% and 92.9% at 1 and 5 years, respectively, with transplant‐free survival rates of 97.7% and 88.1% at 1 and 5 years, respectively. Twelve (27.9%) patients died; two deaths were liver‐related (61 and 134 months postdiagnosis), three deaths were due to intracranial hemorrhage (all on anticoagulation), and one death resulted from an intraoperative complication of liver transplant surgery. Three deaths were from nonliver‐related issues, whereas the remaining three deaths were of unknown cause due to patients being lost to follow‐up.

Among the 11 patients with MELD scores ≥ 18 at diagnosis, 1 died due to hepatopulmonary syndrome at 134 months postdiagnosis, 2 died from intracranial hemorrhage, and 2 died from nonliver causes. One required an LT at 120 months for decompensated liver disease, and four were alive at the end of the study period. One patient was lost to follow‐up.

Of the 22 patients with MELD scores ≤ 17, 15 were alive at the end of the study period, 1 of whom underwent an LT at 48 months. One patient died from liver disease at 61 months postdiagnosis (with no previous TIPS/DIPS), one died of ICH while on anticoagulation, two died of nonliver related causes, one died from an unknown cause, and two were lost to follow‐up. Ten patients had insufficient data to calculate their MELD scores.

## 4. Discussion

The two most pertinent findings from this study are the importance of underlying MPN in patients presenting with primary BCS, and that early radiological intervention may yield favorable transplant‐free survival outcomes.

The incidence of MPN in our cohort was 62.5% among tested patients, which was a higher rate than reported in previous meta‐analyses by Smalberg et al. (41%) [4] and Qi et al. (37%) [20]. This may be explained by the higher rate of testing for MPN with the JAK2V617F mutation in our cohort (79%) compared with 40% by Smalberg et al. [4]. Further, not all JAK2V617F‐negative patients included in Qi et al. [20] underwent bone marrow biopsy testing, where some MPN patients may have been overlooked in the absence of JAK2V617F mutation. The true incidence of MPN within our cohort may still be underestimated, as JAK2V617F and CALR mutation testing only became widely available during the latter half of our study period. Among 12 patients tested for the CALR mutation, two were found to be positive (16.7%), which is a similar rate reported in a previous meta‐analysis [21]. The CALR mutation should be further explored in future studies given its reportedly increasing prevalence in JAK2V617F‐negative MPN [21], compared with previous suggestions of its rarity in BCS/splanchnic vein thrombosis [22–24]. A French group suggests that patients that are JAK2V617F‐negative with a platelet count of >200 × 10^9^/L and a spleen height ≥ 16 cm should undergo CALR mutation testing [25]. Other molecular mutations in MPN such as MPL and JAK2exon12 were not routinely tested in our cohort due to their rarity in BCS [4, 26].

MPN is typically diagnosed following abnormalities in FBE; however, delayed diagnoses are often due to unremarkable biochemistry during early disease. Twenty percentage of our cohort presented with normal FBEs, which is consistent with previous reports [26–28]. Smalberg et al. report that 41% of patients with latent MPN eventually manifest laboratory features of MPN, with a range of 0.7–7 years following JAK2V617F positivity. By comparison, patients in our cohort had MPN diagnosed between 0 and 150 months after BCS diagnosis.

Exogenous estrogen therapy is another common risk factor; however, previous studies suggest it as an associated cofactor when other causative factors are present [1, 29]. In our cohort, 8 out of 27 female BCS patients (29.6%) had estrogen therapy, 7 of whom also had an additional risk factor (MPN or another procoagulant state).

All patients in our study promptly commenced long‐term anticoagulation therapy upon BCS diagnosis. Patients were anticoagulated with either LMWH/enoxaparin, warfarin, or DOAC (apixaban or rivaroxaban), according to international guidelines [9, 10, 30–32]. However, long‐term anticoagulation accrues bleeding risk, and consequently, eight patients in our cohort had major bleeding episodes while on therapeutic anticoagulation. Three bleeding events were fatal intracranial hemorrhages. It may be possible to switch from enoxaparin or warfarin to antiplatelet monotherapy in BCS‐MPN patients with TIPS patency and stabilized peripheral blood counts at 3 years, given that the likely underlying pathophysiology of MPN‐driven BCS involves platelet hyperactivity [33]. However, it is crucial to consider that splenomegaly from portal hypertension may result in splenic platelet pooling where the true circulating platelet count may not be represented in laboratory assays [34].

During this study, DOACs had the lowest rate of TIPS dysfunction. Fourteen patients on DOACs had two episodes of TIPS dysfunction requiring intervention, whereas 37 patients on warfarin had 72 episodes, and 19 patients on LMWH/enoxaparin had 32 episodes. An Austrian multicenter study observed similar rates, where 16 of 22 BCS patients (72.7%) either achieved complete or ongoing response with DOAC treatment [35]. Other studies report comparable rates of efficacy and bleeding complications in DOACs compared with vitamin K antagonists and LMWH during treatment of BCS [36–39]. However, the true efficacy of each anticoagulant could not be determined from this study as most patients trialed multiple agents sequentially; the transition to DOACs from other agents were for various indications at different timepoints. Thus, randomized trials are needed for a comparative analysis between the efficacy of these agents.

Twenty‐three patients (53.5%) received radiological intervention with anticoagulation as primary treatment, including 17 patients (39.5%) who had a TIPS/DIPS inserted during their initial presentation. However, out of 26 patients that did not have TIPS/DIPS as primary treatment, over half (57.7%) had clinical progression of BCS requiring rescue TIPS/DIPS as secondary treatment. Previous single‐center studies have also adopted similar treatment algorithms with early radiological intervention, achieving good survival and transplant‐free rates [40, 41]. Both patients that died of liver‐related causes in our study did not have radiological intervention on presentation. One patient was managed solely with anticoagulation and died at 61 months due to complications of chronic liver disease. The other having delayed TIPS at 123 months but subsequently died of hepatopulmonary syndrome at 134 months. We postulate that the poor outcomes in these patients may be related to delayed radiological intervention.

Our cohort′s median Rotterdam score was 1.13 (range 0.07–2.11), which was comparable with previous studies of BCS cohorts [7, 12]. Sixteen patients were identified as Rotterdam Class I, 18 as Class II, and 5 as Class III, where the predicted survival rates at 5 years were 89% (95% confidence interval [CI] 79%–99%) for Class I, 74% (95% CI 65%–83%) for Class II, and 42% (95% CI 28%–56%) for Class III [1, 13]. Our cohort outperformed the predicted survival rates of the Rotterdam score, with 100% survival at 1 year and 92.9% at 5 years (median follow‐up 134 months).

There were four patients (9.3%) requiring LT in our study. The LT rate was lower compared with published studies following traditional stepwise management, where 12.7% [8] and 42% [1] of patients in those cohorts required LT, respectively. A multicenter prospective study by Seijo et al. [8] reported long‐term outcomes of a standardized stepwise approach in a cohort of 157 BCS patients, where 20 of the 69 patients (29%) that did not receive any invasive management died. The other 88 patients (56%) received invasive management, where 50 patients had TIPS/DIPS as the first‐line intervention and 12 required secondary TIPS/DIPS due to failed angioplasty/thrombolysis. Twenty patients in this study required LT and 36 patients died overall (12 [33.3%] of which were due to liver failure) [8]. In comparison, other single‐center studies report very low to zero LT rates when opting for first‐line radiological intervention [40, 41].

Our findings suggest that early and proactive radiological intervention for primary BCS appears safe and may be associated with excellent long‐term outcomes. The vigilant post‐TIPS/DIPS surveillance program facilitated early identification and correction of any TIPS/DIPS dysfunction. Concurrent management from both hepatology and hematology teams is vital for patients with BCS. These patients should undergo risk factor screening and early aggressive control of hematocrit and peripheral platelet counts if found to have underlying MPN, in addition to immediate initiation of therapeutic anticoagulation. Effective anticoagulation must be ensured even in mild disease not indicating invasive management, given that most patients with clinical symptoms have stenosis in at least two hepatic veins, where suboptimal therapy may lead to rapid hepatic remodeling.

This study has several limitations. Firstly, the small patient cohort did not permit robust multivariable analyses, thus limiting the capacity to infer causal relationships from the data. The decision for early escalated TIPS/DIPS and subsequent anticoagulation regimes were made on an individual basis and this potential selection bias limit the generalizability of the results. We also acknowledge that lower volume centers may not have the specialist expertise or capacity to conduct these escalated practices. Further, the study only includes the PTFE covered stent experiences, thus it is unclear whether other confounding variables—such as advancements in radiological techniques, hematological interventions, and clinical practices over time—may have affected the observed outcomes. Despite these limitations, the analysis provides long‐term safety and efficacy data of early radiological intervention in BCS and offers a framework for future studies to explore early invasive management in comparison with current guideline‐directed algorithms.

## 5. Conclusion

Our single‐center experience suggests MPN as an important underlying cause of primary BCS, particularly JAK2V617F‐positive MPN. We advocate that all BCS patients should undergo hematological screening for the JAK2V617F gene mutation (and CALR mutation testing if JAK2‐negative), with bone marrow biopsy if testing for both mutations is negative in the setting of abnormal peripheral blood counts. We suggest that early radiological intervention should be considered in primary BCS management as it is possible to achieve excellent survival and transplant‐free outcomes with early angioplasty/stenting and TIPS. DOACs were used in our cohort without significant safety concerns, but their comparative efficacy cannot be determined from these data. Further large prospective studies are required to validate this model compared with the traditional “step‐up” management.

## Funding

No funding was received for this manuscript.

## Conflicts of Interest

The authors declare no conflicts of interest.

## Data Availability

The data that support the findings of this study are available on request from the corresponding author. The data are not publicly available due to privacy or ethical restrictions.
